# Vocalisation Repertoire at the End of the First Year of Life: An Exploratory Comparison of Rett Syndrome and Typical Development

**DOI:** 10.1007/s10882-022-09837-w

**Published:** 2022-03-08

**Authors:** Katrin D. Bartl-Pokorny, Florian B. Pokorny, Dunia Garrido, Björn W. Schuller, Dajie Zhang, Peter B. Marschik

**Affiliations:** 1 iDN – interdisciplinary Developmental Neuroscience, Division of Phoniatrics, Medical University of Graz, Graz, Austria; 2 EIHW – Chair of Embedded Intelligence for Health Care and Wellbeing, University of Augsburg, Augsburg, Germany; 3 Mind, Brain, and Behaviour Research Centre, University of Granada, Granada, Spain; 4 GLAM – Group on Language, Audio, & Music, Department of Computing, Imperial College London, London, UK; 5 Child and Adolescent Psychiatry and Psychotherapy, Systemic Ethology and Developmental Science, University Medical Center Göttingen, Georg-August University Göttingen, Göttingen, Germany; 6 Leibniz ScienceCampus Primate Cognition, Göttingen, Germany; 7 Center of Neurodevelopmental Disorders (KIND), Department of Women’s and Children’s Health, Karolinska Institutet, Stockholm, Sweden

**Keywords:** Canonical babbling, Early vocalisations, Infant, Late detected developmental disorders, Rett syndrome, Speech-language impairment

## Abstract

Rett syndrome (RTT) is a rare, late detected developmental disorder associated with severe deficits in the speech-language domain. Despite a few reports about atypicalities in the speech-language development of infants and toddlers with RTT, a detailed analysis of the pre-linguistic vocalisation repertoire of infants with RTT is yet missing. Based on home video recordings, we analysed the vocalisations between 9 and 11 months of age of three female infants with typical RTT and compared them to three age-matched typically developing (TD) female controls. The video material of the infants had a total duration of 424 min with 1655 infant vocalisations. For each month, we (1) calculated the infants’ canonical babbling ratios with CBR^UTTER^, i.e., the ratio of number of utterances containing canonical syllables to total number of utterances, and (2) classified their pre-linguistic vocalisations in three non-canonical and four canonical vocalisation subtypes. All infants achieved the milestone of canonical babbling at 9 months of age according to their canonical babbling ratios, i.e. CBR^UTTER^ ≥ 0.15. We revealed overall lower CBRs^UTTER^ and a lower proportion of canonical pre-linguistic vocalisations consisting of well-formed sounds that could serve as parts of target-language words for the RTT group compared to the TD group. Further studies with more data from individuals with RTT are needed to study the atypicalities in the pre-linguistic vocalisation repertoire which may portend the later deficits in spoken language that are characteristic features of RTT.

## Introduction

Rett syndrome (RTT; OMIM 312,750) is a severe developmental disorder mostly caused by de novo mutations in the *MECP2* (methyl-CpG binding protein 2) gene on the long arm of the X chromosome (Xq28) (Amir et al., 1999; Zoghbi, 2005). RTT has a prevalence of approximately 1 of 10,000 live female births (Hagberg, 1985; Laurvick et al., 2006); male individuals with RTT are very rare (Christen & Hanefeld, 1995). Clinical diagnosis of RTT is based on four core consensus criteria (Neul et al., 2010): 1. partial or complete regression (i.e., loss) of already acquired purposeful hand skills, 2. regression of already acquired spoken language, 3. gait abnormalities, and 4. stereotypic hand movements. Most individuals with RTT have an onset of regression between 12 and 18 months of age (Burford et al., 2003; Einspieler & Marschik, 2019; Lee et al., 2013). The classic form of RTT is currently diagnosed at a mean age of 2.7 years (Tarquinio et al., 2015). Like for other late detected developmental disorders, the late diagnosis of RTT hinders the implementation of early, individually tailored interventions for affected children. This motivates research on early development to promote earlier identification of affected individuals. As deficits in the speech-language domain compose a core characteristic of RTT, a thorough investigation of the pre-linguistic speech-language development may reveal early signs that portend later associated impairments.

Pre-linguistic vocalisations are typically produced throughout the first year of life, preceding the first referential words. Based on vocal data of typically developing (TD) children, specific vocalisation schemes have been developed with the aim to phonetically categorise these pre-linguistic vocalisations (Nathani et al., 2006; Papousek, 1994). The schemes define a number of vocalisation patterns and related studies investigated the onset and the proportional use of these patterns in the vocalisation repertoires of infants (Nathani et al., 2006; Oller, 1980; Papousek, 1994; Stark, 1980, 1981). One of the most salient pre-linguistic vocalisation patterns is canonical babbling, typically emerging between 5 and 10 months of age (Lang et al., 2019; Morgan & Wren, 2018; Oller, 1980, 2000). Infants produce canonical syllables by combining consonant(-like) sounds and vowel(-like) sounds with fast formant transitions between consonant and vowel (Oller, 2000; Oller et al., 1999; Papousek, 1994). These canonical syllables often occur in series (e.g., /babu/, /mamama/, /dadama/) (Nathani et al., 2006). The ability of an infant to produce canonical syllables and practice them in various combinations is crucial for the production of referential words as these usually consist of canonical syllables (Lee et al., 2018; Morgan & Wren, 2018; Oller et al., 1998).

Studies varied in their definitions of canonical babbling and in their parameters chosen to define the onset of canonical babbling (Lang et al., 2019; Molemans et al., 2012; Roche et al., 2018). Some studies defined canonical babbling to be acquired with the first occurrence of a canonical syllable or two combined canonical syllables (Bartl-Pokorny et al., 2013; Marschik et al., 2013; Schramm et al., 2009), whereas others defined a certain canonical babbling threshold (Lang et al., 2020; Molemans et al., 2012; Oller & Eilers, 1988; Oller et al., 1994; Patten et al., 2014). A widely used measure to determine the onset of canonical babbling as well as the proportion of this verbal pattern in an infant’s vocalisation repertoire at a given age is the canonical babbling ratio (CBR) (Oller & Eilers, 1988). It was originally defined as the ratio of canonical syllables to total number of utterances (CBR^utt^) (Oller & Eilers, 1988). In the last 30 years, a number of different CBR measures were proposed; for an overview see Molemans and colleagues (2012). These differ mainly in the exact definition of a canonical syllable and in whether the number of canonical syllables is divided by the total number of syllables or by the total number of utterances (Lang et al., 2019). The only CBR measure that can be applied without counting the number of canonical syllables is CBR^UTTER^, the most recent CBR measure (Nyman & Lohmander, 2018). CBR^UTTER^ is defined as the ratio of number of utterances containing canonical syllables to total number of utterances. Nyman and Lohmander (2018) and Lang and colleagues (2020) found high correlations between CBR^UTTER^ and other CBR measures, yet CBR^UTTER^ is less time-consuming to obtain. Thus, we decided to use CBR^UTTER^ for the current study. A child is regarded as having reached the canonical babbling milestone if his or her CBR is found to be higher than a defined threshold. For CBR^UTTER^, the threshold is set at 0.15 (Nyman & Lohmander, 2018).

Irrespective of different criteria used to define the onset of canonical babbling, not meeting this important milestone at 10 months of age has been discussed as early indicator of atypical speech-language development (Lang et al., 2019; Lohmander et al., 2017; Oller et al., 1998, 1999; Yankowitz et al., 2019). For example, infants with profound hearing impairment (Löfkvist et al., 2020; Schauwers et al., 2004), Down syndrome (Lynch et al., 1995), autism spectrum disorder (Patten et al., 2014), or fragile X syndrome (Belardi et al., 2017) were found to have lower CBRs in comparison to TD infants and/or to fail to reach the defined CBR threshold for the onset of canonical babbling in time.

The reported deviances in CBR of infants with developmental disorders lead to the assumption that deviances in CBR could also be present in infants with other late detected developmental disorders that are associated with deficits in the speech-language domain, such as RTT. To the best of our knowledge, the CBR has not been investigated so far for infants with RTT. Still, studies analysing home videos of infants with RTT indicated early speech-language peculiarities already in the pre-regression phase, including deviant canonical babbling (Bartl-Pokorny et al., 2013; Einspieler & Marschik, 2019; Einspieler et al., 2014; Marschik et al., 2011, 2013, 2014a, b; Pokorny et al., 2018; Townend et al., 2015). For example, Marschik and colleagues (2013) observed canonical babbling (as defined by at least one occurrence of two successive consonant–vowel combinations; e.g., /baba/) in only 5 out of the 10 children with RTT during the first two years of life. Bartl-Pokorny and colleagues (2013) reported that none of the sampled six 9– to 12-month old infants with RTT was observed to use canonical babbling for communicative purposes such as directing attention of self or requesting an object. These findings, together with reports of individuals with other developmental disorders not achieving the canonical babbling milestone in time, led us to explore pre-linguistic vocalisations of individuals with RTT in more detail at an age (i.e., 9 to 11 months) when TD infants are expected to achieve the milestone of canonical babbling. With the present study, we aimed to provide for the first time a meticulous comparison of the vocalisation repertoires of 9- to 11-month old infants with RTT and TD infants. For this, we (i) provided the CBRs of the infants and (ii) classified the infants’ non-canonical and canonical pre-linguistic vocalisations in subtypes. We hypothesised that infants with RTT and TD infants differ in their CBRs and that infants with RTT and TD infants differ in the composition of their non-canonical and canonical pre-linguistic vocalisation repertoires. The aim of this exploratory study was to provide a starting point towards a better understanding of the pre-linguistic vocalisations of individuals with RTT.

## Materials and Methods

We used a retrospective video analysis approach to compare the vocalisations of infants later diagnosed with RTT and of TD infants. The study was approved by the local research ethics committee.

### Participants

For the present study, we included data of infants with RTT and of TD infants from our database. Inclusion criteria for the participants with RTT were: (a) a confirmed clinical diagnosis of typical RTT, (b) infant was brought up in a monolingual German-speaking family, (c) audio-video material was available between 9 and 11 months (see also "Material"). Following these inclusion criteria, the available participants were three females with RTT (RTT1–RTT3). Genetic testing revealed the following pathogenic *MECP2* mutations: p.R168X for RTT1, p.F157L for RTT2, and p.R106W for RTT3. The infants with RTT were matched with three TD infants (TD1–TD3) for gender, age at time of recording of video material, and family language. All infants were singletons and were born at term.

### Material

Analysis was based on home video recordings that were taken by the infants’ parents during daily routines or special family events. At the time of recording, the parents of the participants with RTT were not aware of their children’s medical condition. The material was either provided by the participants themselves (TD group) or their parents (RTT group), who gave their informed consent for analysis of the data for research purposes and for publication of the results. In the present study, we included all available home video material taken from the participants when they were 9 to 11 months old, to focus on the pre-linguistic period in which TD infants are expected to have achieved the milestone of canonical babbling (Lang et al., 2019; Lohmander et al., 2017; Oller et al., 1998, 1999; Yankowitz et al., 2019). The total duration of the included home video material was 424 min (RTT1: 191 min, RTT2: 81 min, RTT3: 14 min, TD1: 61 min, TD2: 44 min, and TD3: 33 min). The videos of the TD group were recorded in the years 1991/1992 (TD1), 1988 (TD2), and 1998 (TD3). The videos of the RTT group were recorded between the years 1994 and 2003.

A trained research assistant blind to the purpose of the project prepared the video material for analysis with the video coding system Noldus Observer XT (https://www.noldus.com): First, the videos were annotated for scenes showing the infants in settings ‘with social interaction’ vs ‘without social interaction’. Second, the videos were marked for infant vocalisations by setting ‘start’ and ‘stop’ tags. A breath-group criterion was used to segment vocalisations, i.e., segment boundaries were set in case of ingressive breathing (Nathani & Oller, 2001). Inspiratory sounds were defined as regular parts of a vocalisation and did not mark segment boundaries. Vegetative sounds (e.g., breathing sounds, sneezes, hiccups) were not segmented and were excluded from further analysis. Third, each vocalisation was exported as a separate audio clip, which was labelled with a randomly assigned numeric code. The audio clips prepared for subsequent coding did not include information on participant ID, age, and developmental outcome. As several studies have suggested that volubility and vocalisation patterns such as canonical babbling may be sensitive to social circumstances, e.g., interaction vs no interaction with caregiver (Goldstein & Schwade, 2008; Iyer et al., 2016; Lee et al., 2018), and for better comparability regarding recording situation, only those vocalisations produced in the ‘with social interaction’ settings (i.e., 96% of the entire vocalisations) were selected for further analyses. All segmented vocalisations were double-checked and verified for segment boundaries by the second author. The final dataset for analysis consisted of 1655 vocalisations (RTT1: 735, RTT2: 166, RTT3: 121, TD1: 197, TD2: 240, and TD3: 196). Vocalisation duration ranged from 0.28 to 12.26 s (Mean = 1.64, SD = 1.12). The shortest vocalisation, uttered by TD3 at 10 months of age, was a single vowel-like sound. The longest vocalisation, uttered by TD2 at 11 months of age, was a combination of several consonant-like and vowel-like sounds interspersed with short pauses without ingressive breathing. For RTT1 we had vocalisation data available for each of the three months of interest; for RTT2 data were available for 9 and 10 months only, for RTT 3 data were available for 9 months only, and for TD1, TD2, and TD3 we had vocalisation data available for each of the three months.

### Vocalisation Classification

Vocalisations that were produced in a neutral mood, referred to as pre-linguistic vocalisations (PLVs) were classified according to the vocalisation scheme presented in Table 1. The scheme was similar to the ‘Stark Assessment of Early Vocal Development-Revised’ (SAEVD-R) (Nathani et al., 2006) with alterations for our study purposes. We included vocalisation types that are characteristic for the investigated age. In particular, the following alterations compared to SAEVD-R were carried out to minutely represent the infants’ age-specific vocalisation repertoires and their stratified complexity: 1. We classified non-canonical vocalisations into three subtypes, i.e. PLV1–PLV3, instead of annotating all Level 1 to Level 3 vocalisation types defined by the SAEVD-R which are targeted on capturing the vocalisation repertoire of younger infants compared to our target group; 2. For the canonical realisations with 2 sounds, we defined a vocalisation subtype for the combinations of 1 vowel(-like) and 1 consonant(-like) sound with a rapid formant transition between the sounds, and with *at least 1* of these sounds *not* conforming to the target language (i.e. PLV4), as a contrast to PLV5, which consists of *both* sounds conforming to the target language. Vowel-like and consonant-like sounds are not yet well-formed vowels and consonants that could serve as parts of target-language words (Oller, 2000) and therefore cannot be accurately transcribed with the International Phonetic Alphabet (IPA), a widely used system to transcribe speech sounds (International Phonetic Association, 2018); 3. For the canonical vocalisations with 3 or more sounds, we added the category PLV6, with *at least* 1 of the sounds not conforming to the target language, and PLV7, with *all* the sounds conforming to the target language. Note that in SAEVD-R, ‘VC’ syllables assigned to PLV5 in our scheme and all vocalisations assigned to PLV7 are defined identically as ‘complex syllables’ (CMPX), regardless of the number of sounds included. In our scheme, we separated the vocalisations with two sounds from those with three or more sounds marking different vocal complexities; 4. We excluded the subtype for consonant(-like)-vowel(-like) combinations with prolonged formant transitions (classified as ‘marginal babbling’ in SAEVD-R) as the corpus for the present study did not contain such realisations.

Each vocalisation was first assigned to one of the three vocalisation types (i) pleasure (i.e., laughing, pleasure bursts), (ii) distress (i.e., fussing, crying), or (iii) PLV. Each PLV was then assigned to one of seven mutually exclusive vocalisation subtypes (i.e., PLV1–PLV7; see Table 1). Vocalisations assigned to PLV1, PLV2, or PLV3 did not contain canonical syllables while vocalisations assigned to PLV4, PLV5, PLV6, or PLV7 contained canonical syllables (Table 1). Vocalisation segmentation according to a breath-group criterion (see "Material") may result in two or more segments within one PLV (separated by pauses without ingressive breathing). Consequently, a PLV may include segments of different vocalisation subtypes, e.g., both a single vowel-like sound (i.e., PLV1) and a vowel-consonant combination (i.e., PLV5). If so, the PLV was assigned to the highest vocalisation subtype index included, ascending from PLV1 to PLV7. For example, a PLV including both a PLV1-segment and a PLV5-segment was coded as PLV5. Our approach to assign a PLV to the highest vocalisation subtype index included allowed us to capture all canonical babbling occurrences in the corpus as all canonical vocalisation subtypes (i.e., PLV4–PLV7) have higher subtype indexes than the non-canonical vocalisation subtypes (i.e., PLV1–PLV3).

Three coders (first author, second author, third author) independently annotated all 1655 vocalisations according to the scheme (Table 1). This annotation procedure resulted in a majority vote (i.e., at least two of the three coders agreed on the classification) for 1533 vocalisations, determining the final vocalisation type and subtype annotation for the respective vocalisations. The remaining 122 vocalisations for which no majority vote was available (7.4% of all vocalisations) were discussed within the team until consensus on the classification was achieved.

### Analysis

We performed the following three steps to meet our research goals: First, we identified the number of vocalisations assigned to the respective vocalisation types (i.e., pleasure, distress, PLV) and the PLV subtypes (i.e., PLV1-PLV7) for each infant and month of age. Second, we computed the distribution (as percentage) of PLV1-PLV7. Third, we calculated the infants’ CBRs by using CBR^UTTER^ (Nyman & Lohmander, 2018). The CBR^UTTER^ can be easily derived from our vocalisation subtype analysis by dividing the sum of PLVs containing canonical syllables (i.e., PLV4-PLV7) by the total number of PLVs of the respective infant. The threshold for reaching the canonical babbling milestone was CBR^UTTER^ ≥ 0.15, as defined by Nyman and Lohmander (2018).

## Results

**Fig. 1:**
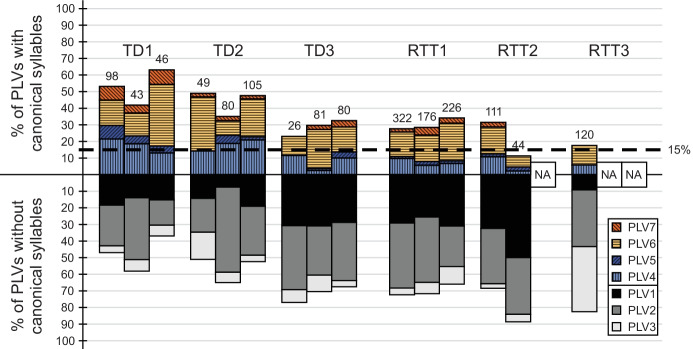
Distribution (as percentage) of the pre-linguistic vocalisation subtypes (PLV1–PLV7) produced by the participants at 9 months of age (left bar), 10 months (middle bar), and 11 months (right bar). Each bar represents the total PLV repertoire (hundred-percent) analysed for the month. Numbers on top of the bars indicate the number of PLVs available for analysis of the month. The dashed line indicates the 0.15 threshold of reaching the canonical babbling milestone. PLV = pre-linguistic vocalisation; NA = no data available for the respective month

The vast majority of all infants’ vocalisations at 9, 10, and 11 months were PLVs (see Table 2). Figure 1 illustrates the distribution (as percentage) of PLV1–PLV7 produced per infant and month. All infants produced both canonical (coloured and ruled bars, Fig. 1) and non-canonical (grey shaded bars, Fig. 1) PLVs, the latter forming the major component of most infants’ vocalisation repertoires. As indicated by the dashed line in Fig. 1, all six infants achieved the canonical babbling milestone by 9 months of age (i.e., CBR^UTTER^ ≥ 0.15). Figure 1 presents a generally higher proportion of canonical PLVs in the TD than in the RTT group. The CBRs^UTTER^ of the infants varied from month to month (for exact CBR^UTTER^ values please refer to bottom of Table 2). Despite the variation, TD1 consistently had the highest CBR^UTTER^ of the six infants from 9 to 11 months, followed by TD2. The lowest observed CBRs^UTTER^ of TD1 and TD2 (i.e., 0.42 and 0.35 at 10 months) were higher than the highest CBRs^UTTER^ of the remaining four infants (bottom of Table 2) and were considerably higher than the threshold of 0.15. The lowest CBRs^UTTER^ in this sample were observed in the RTT group (i.e., RTT3 at 9 months, 0.18; and RTT2 at 10 months, 0.11). TD3 and RTT1 demonstrated considerable similarities concerning their CBRs^UTTER^ in all three months (Fig. 1). A strong reduction in CBR^UTTER^ was only observed for RTT2 (i.e., from 0.32 at 9 months to 0.11 at 10 months; Table 2), who was also the only infant who ever presented a CBR^UTTER^ lower than 0.15.

As shown in Fig. 1, the majority of canonical PLVs of all infants did not conform to the target language (i.e., vocalisations of PLV4 and PLV6). Except for RTT3, all infants were observed to produce canonical PLVs conforming to the target language (i.e., vocalisations of PLV5 and PLV7). Figure 1 presents a generally higher proportion of canonical PLVs conforming to the target language in the TD than in the RTT group (for exact values please refer to bottom of Table 2). TD1, the infant with the highest CBR^UTTER^ across all months, was the one who produced the highest proportion of PLV5 and PLV7.

Besides their comparable CBRs^UTTER^, TD3 and RTT1 demonstrated comparable distributions of both their canonical and non-canonical PLV subtypes across time. RTT3 demonstrated a distinct distribution of the non-canonical PLV subtypes at 9 months, producing a much higher proportion of PLV3 than the other infants.

## Discussion

In this study, using retrospective video analysis, we investigated and compared the pre-linguistic vocal behaviours of three infants later diagnosed with RTT with their age-matched TD peers. Following findings deserve special comments.

### Canonical Babbling Ratio

Our results show that all infants with RTT and all TD infants reached the canonical babbling milestone by 9 months, i.e. CBRs^UTTER^ ≥ 0.15 (Table 2, Fig. 1). As canonical babbling is achieved by most children with typical outcome when they are between 5 and 10 months of age (Lang et al., 2019; Morgan & Wren, 2018; Oller, 1980), the three infants with RTT met the canonical babbling milestone in time. This finding confirms that reaching the canonical babbling milestone in time alone is not sufficient to predict typical speech-language development (Lang et al., 2021; Oller et al., 1998, 1999).

Even though all infants met the canonical babbling milestone in time, we found differences in the CBRs^UTTER^ between the RTT and the TD group: Despite the varying and partly small number of available data per month, the TD group had overall considerably higher CBRs^UTTER^ than the RTT group. The TD infants clearly and consistently exceeded the canonical babbling threshold (0.15) by producing a high proportion of canonical vocalisations. TD1 and TD2 had the highest CBRs^UTTER^ of all infants in all three months, whereas the lowest CBRs^UTTER^ were observed in RTT2 and RTT3. Our findings are in line with the studies focusing on other developmental disorders: Lower CBRs in comparison with TD infants were previously reported for infants with autism spectrum disorder (Patten et al., 2014) and fragile X syndrome (Belardi et al., 2017). As these disorders are associated with later speech-language impairments, our findings support the body of evidence that early signs may emerge and be detectable already during the canonical babbling period forecasting later more apparent language deficits.

Notably, the amount of available data does not seem to be associated with the infants’ proportion of canonical babbling. For example, although all TD infants had in all months far lower quantity of available data (i.e., number of PLVs) than RTT1, their CBRs^UTTER^ were much higher or at least comparable to the ones presented by RTT1. Similarly, even though RTT3 had more PLVs in the 9 month’s data (N = 120) than any TD infant had at any single month, her CBR^UTTER^ was among the lowest observed. As a contrast, for TD3 in the 9-month’s observation, although only 26 PLVs were available, the CBR^UTTER^ was still at 0.23. We cannot rule out the possibility, however, that if we had more data available, the profile might change. Especially for infants who may reveal atypical development, comprehensive data may be crucial to reflect their true capacities and potential. Balanced and adequate amount of data is desired for any empirical study, yet hardly feasible for retrospective natural observations. Nevertheless, our data demonstrated that the data quantity does not account for the quality and complexity of the infants’ vocalisation repertoire. A greater quantity of vocalisations does not necessarily increase the CBR^UTTER^ and even a limited amount of data could capture the achievement of the canonical babbling milestone, at least for infants with typical development.

In our study, we analysed the CBRs^UTTER^ per month. As the data amount and the recording settings of each infant were heterogeneous, the CBRs^UTTER^ of both the infants with RTT and the TD infants varied naturally from month to month. Although previous studies reported a general increase of canonical babbling following its onset, fluctuations in the proportional use of vocalisation subtypes are common in typical infant development (Morgan & Wren, 2018; Nathani et al., 2006; Oller et al., 1994). Therefore, it is not surprising to observe, for example, lower CBRs^UTTER^ at 10 months compared to 9 months for TD1 and TD2, given that both CBRs^UTTER^ remained at a high level (0.35 and 0.42, respectively). This is in line with previous longitudinal studies on canonical babbling, which found that once TD infants reached the canonical babbling stage, their CBR usually continued to stay above the canonical babbling threshold at the subsequent assessment points (Lynch et al., 1995; Oller et al., 1994). In comparison, the onset of canonical babbling in infants with Down syndrome (Lynch et al., 1995), preterm infants (Oller et al., 1994), and infants with hearing loss (Nathani et al., 2007; Oller & Eilers, 1988) was reported to be less stable: In the consecutive assessments after the onset of canonical babbling, these infants were less likely to maintain a CBR above the threshold. This is exactly the case also in our findings: Of three infants revealing a downward fluctuation from 9 to 10 months, RTT2 was the only one with the CBR^UTTER^ dropping below the threshold (< 0.15), whereas TD1 and TD2 remained a high level of CBR^UTTER^ (see discussion above and Table 2). Again, note that TD1 and RTT2 had comparable amounts of available data for the 10-month’s analysis, suggesting that their different CBRs^UTTER^ cannot be explained by the quantity of PLVs. Unfortunately, we had no data available for RTT2 at an older age to track her CBR^UTTER^ and her general developmental profile. It raises an interesting question whether her significant reduction of CBR^UTTER^ could reflect the onset of her regression. As reported by Tarquinio and colleagues (2015) with a natural history study collecting caregiver reports (based on baby books, photos, and videos), *MECP2* testing dates, and clinician notes, a regression of babbling was found in 37.9% of 869 participants of the RTT cohort. Future studies are needed to investigate whether a reduction of CBR^UTTER^, if presented, coincides with the onset of regression in individuals with RTT.

RTT1 was the only infant of our RTT sample from whom we had data in all targeted months. Contrary to RTT2, RTT1 did not display a downward fluctuation during the observation period. Indeed, her profile of PLVs was similar to an infant with typical development, i.e., TD3 (please see further discussion on this topic in "Overlaps Between Typical and Atypical Development"). As most individuals with RTT are reported to experience regression after their first birthday (Burford et al., 2003; Einspieler & Marschik, 2019; Lee et al., 2013), RTT1’s profile in this specific pre-linguistic domain may reflect the pre-regressional pathway that has often been interpreted as inconspicuous until the end of the first year of life. It would be interesting to see whether comparably higher proficiency in pre-linguistic skills may also relate to a more favourable language outcome in the study group and in a larger sample of individuals with RTT. Unfortunately, we do not have data at the moment to further examine this issue.

### Pre-linguistic Vocalisation Subtypes

In addition to the CBR^UTTER^, we analysed for the first time the structure of the canonical PLVs (i.e., PLV4–PLV7) and non-canonical PLVs (i.e., PLV1–PLV3) of infants with RTT and compared it to TD infants. The majority of canonical PLVs of all infants did not conform to the target language (i.e., vocalisations of PLV4 and PLV6). This is consistent with the general knowledge that a great proportion of infant sounds are not well-formed vowels and consonants that could serve as parts of target-language words (Oller, 2000). When comparing the RTT and the TD cases, we found that all three infants with TD and two of three infants with RTT used target-language canonical PLVs (i.e., vocalisations of PLV5 and PLV7). As a group, the TD infants had a higher proportion of target-language canonical PLVs compared to the infants with RTT (Table 2). It is noteworthy that the infant TD1 with the highest CBRs^UTTER^ in the sample produced the highest proportion of target-language canonical PLVs (i.e., PLV5 and PLV7; Fig. 1), whereas the only infant without target-language canonical PLVs, i.e. RTT3, was among the two infants with the lowest CBRs^UTTER^. However, we only had data available for RTT3 at 9 months of age. For comparison, we also did not observe target-language canonical PLVs for TD3 at 9 months, yet in the data recorded later. Nathani and colleagues (2006) found that combinations of well-formed consonants and vowels with rapid formant transitions between them, i.e., vocalisations of Level 4 and Level 5 according to the SAEVD-R, occurred only to a small proportion before 9 months of age, but increased from 9–12 months onwards and especially in the second year of life in TD children. Following this observation, it would be interesting for future studies to track the progression of target-language canonical PLVs in children with RTT for a longer period of time. Besides the fact that RTT3 just met the canonical babbling milestone (CBR^UTTER^ = 0.18) and was the only infant without target-language canonical PLVs in her data, she demonstrated a distinct distribution of the non-canonical PLV subtypes, producing a much higher proportion of PLV3 (i.e., a combination of two or more vowel(-like) sounds with a change in pitch, intensity, and/or formants) than the other infants. PLV3 has been regarded as a precursor of canonical babbling as the SAEVD-R (Nathani et al., 2006) classifies vocalisations of PLV3 as ‘marginal babbling’. As we do not have further data for RTT3, we are not able to learn whether a reduction of the use of PLV3 and an increase of canonical PLV subtypes conforming to the target language would take place. Neither could we know from the current data whether such a distinct distribution of the non-canonical PLV subtypes might precede later deviant language development. This issue deserves further exploration with more data from additional individuals with RTT.

### Overlaps Between Typical and Atypical Development

While TD3 and RTT1 presented similar CBR^UTTER^ profiles, TD3 was the poorest vocaliser of the TD infants, and RTT1 the best of the RTT group. Such comparable profiles of RTT and TD infants are expectable since the development of children with typical outcome does not necessarily distinguish in overt behavioural traits from children with atypical outcome in a clear-cut manner. Rather, our observation nicely reflects a natural phenomenon that within a developmental dimension, e.g., language development, the lower end of the continuum of typical development (compromised yet within the normal range) frequently merges with the higher end of that of atypical development, which makes it challenging to identify a true deviation. However, development should never be seen single-dimensionally. With close-meshed observations investigating different aspects of neurofunctional development, across domains and time, atypical signs and profiles (behavioural biomarkers) as well as protective factors will likely to be revealed sooner (Marschik et al., 2017). Notably, given the seemingly similar vocalisation patterns, TD3 increased her proportion of canonical PLVs conforming to the target language (i.e., PLV5 and PLV7) steadily from month to month, while this was not the case for RTT1 (Table 2). Could this suggest that behind the similarities, qualitative differences still exist between their PLVs? Moreover, in the current study, only one aspect of speech-language development, i.e., the subtypes of the PLVs, was compared among the infants. Other methods evaluating infant vocalisations, e.g., acoustic analysis (Pokorny et al., 2016), might help to further examine whether apparently similar vocalisation profiles are genuinely comparable to each other.

### Limitations

The present study is limited by the heterogeneous data quantities and the data recording settings, an inherent restriction in retrospective research and studies in natural settings (Marschik & Einspieler, 2011; Ozonoff et al., 2011; Palomo et al., 2006). The small sample size available prevents us from drawing more general conclusions. Still, our study is a first step and a primer to explore detailed vocalisation profiles and the early speech-language development of infants later diagnosed with RTT, and their differences to the TD peers at this early age. Our findings revealed unignorable differences in pre-linguistic vocalisation repertoires between TD infants and infants with RTT during the typical canonical babbling period that cannot be reduced to the quantity of data. Questions arising from our study deserve further investigation with more data to gain insights into early speech-language development in RTT.

### Concluding Remarks

Our study supports previous studies on the early speech-language development of infants with RTT (Bartl-Pokorny et al., 2013; Einspieler & Marschik, 2019; Marschik et al., 2013, 2014a) and adds new insights into pre-linguistic vocalisation repertoires: All infants achieved the milestone of canonical babbling by 9 months of age according to their CBRs^UTTER^. We revealed overall lower CBRs^UTTER^ and a lower proportion of canonical PLVs conforming to the target language for the RTT group compared to the TD group. Hopefully, our work will trigger more studies to identify subtle atypicalities in the pre-linguistic vocalisations preceding the obvious language impairments that are characteristic of numerous developmental disorders. Observable overlaps between the RTT and the TD infants in our study exemplify that an early identification of developmental deficits based solely on the pre-linguistic vocalisation repertoire is unlikely. Rather, an in-depth understanding of the speech-language capacities of individuals with RTT from early on, together with efforts of investigating other developmental domains as well as comparing RTT to different conditions, will draw us closer to an earlier identification of this otherwise late detected developmental disorder.

**Table 1:**
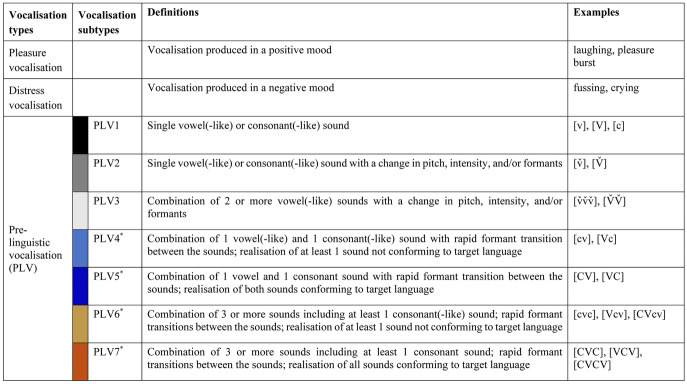
Classification scheme of infant vocalisations. [c]/[v] = consonant-like/vowel-like sound, cannot be accurately transcribed with the International Phonetic Alphabet (IPA) (International Phonetic Association, 2018); [C]/[V] = consonant/vowel, included in the IPA; PLV = pre-linguistic vocalisation, produced in a neutral mood; ˇ = IPA diacritic to indicate an ascending pitch (rising contour); ^*^ = canonical babbling (Oller et al., 2000, 1999)

**Table 2:**
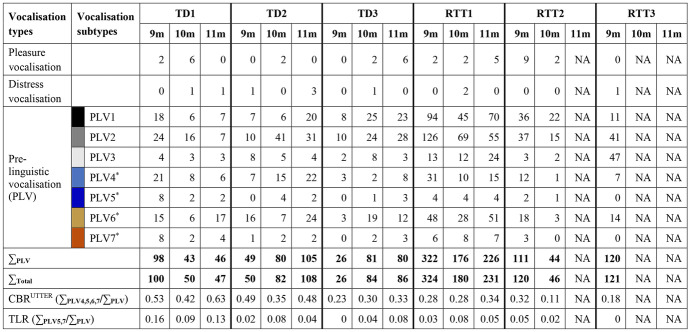
Number of vocalisations assigned to the respective vocalisation types and subtypes for the participants at 9, 10, and 11 months of age. CBR^UTTER^ = canonical babbling ratio; NA = no data available for the respective month; TLR = ratio of canonical PLVs conforming to the target language; ^*^ = canonical babbling (Oller et al., 2000, 1999)
